# Importance of Non-pharmacological Approaches for Treating Irritable Bowel Syndrome: Mechanisms and Clinical Relevance

**DOI:** 10.3389/fpain.2020.609292

**Published:** 2021-01-21

**Authors:** Albert Orock, Tian Yuan, Beverley Greenwood-Van Meerveld

**Affiliations:** ^1^Oklahoma Center for Neuroscience, University of Oklahoma Health Sciences Center, Oklahoma City, OK, United States; ^2^Oklahoma City VA Health Care System, Oklahoma City, OK, United States; ^3^Department of Physiology, University of Oklahoma Health Sciences Center, Oklahoma City, OK, United States

**Keywords:** pain, environmental enrichment, behavioral therapy, visceral hypersensitivity, stress

## Abstract

Chronic visceral pain represents a major unmet clinical need with the severity of pain ranging from mild to so severe as to prevent individuals from participating in day-to-day activities and detrimentally affecting their quality of life. Although chronic visceral pain can be multifactorial with many different biological and psychological systems contributing to the onset and severity of symptoms, one of the major triggers for visceral pain is the exposure to emotional and physical stress. Chronic visceral pain that is worsened by stress is a hallmark feature of functional gastrointestinal disorders such as irritable bowel syndrome (IBS). Current pharmacological interventions for patients with chronic visceral pain generally lack efficacy and many are fraught with unwanted side effects. Cognitive behavioral therapy (CBT) has emerged as a psychotherapy that shows efficacy at ameliorating stress-induced chronic visceral pain; however, the molecular mechanisms underlying CBT remain incompletely understood. Preclinical studies in experimental models of stress-induced visceral pain employing environmental enrichment (EE) as an animal model surrogate for CBT are unraveling the mechanism by which environmental signals can lead to long-lasting changes in gene expression and behavior. Evidence suggests that EE signaling interacts with stress and nociceptive signaling. This review will (1) critically evaluate the behavioral and molecular changes that lead to chronic pain in IBS, (2) summarize the pharmacological and non-pharmacological approaches used to treat IBS patients, and (3) provide experimental evidence supporting the potential mechanisms by which CBT ameliorates stress-induced visceral pain.

## Introduction

Irritable bowel syndrome (IBS) is a chronic gastrointestinal (GI) disorder that affects about 10–20% of the population of the USA (1). IBS is typically characterized by visceral hypersensitivity that presents as chronic abdominal pain accompanied by abnormal bowel habits such as diarrhea (IBS-D) or constipation (IBS-C) (1, 2), usually without any obvious histological damage. IBS is diagnosed by gastroenterologists using the ROME criteria (ROME IV) (3). The duration and severity of patient symptoms range from mild to severe enough to detrimentally affect the patients' quality of life (4, 5). The exact etiology of IBS is unclear and research has revealed that the cause of the disorder is multifactorial with many different biological and psychological systems contributing to the onset and severity of symptoms. The lack of understanding of the mechanisms behind IBS has caused a paucity in the development of effective pharmacological treatments of IBS. Stress is a significant risk factor for the emergence of chronic visceral pain in IBS and is often comorbid with other mood and anxiety disorders (6–9), suggesting that environmental factors and stimuli can influence visceral pain sensation in IBS.

Most pharmacological therapies to alleviate IBS symptoms show limited efficacy and can cause unwanted side effects (10, 11). Behavioral therapies have been employed to control the symptoms of mood and psychiatric disorders (12–14), and evidence suggests that behavioral therapies may have potential for the treatment of IBS. Cognitive behavioral therapy (CBT) is a form of psychosocial therapy used to change patterns of thought and emotions. CBT has been approved for a number of psychiatric disorders including anxiety, drug abuse, and depression (15–17). Clinical studies suggest that CBT may also be effective in ameliorating visceral pain disorders including IBS (18, 19). Unfortunately, CBT is not widely available for IBS patients due to a paucity of trained specialists, as well as the duration of treatment sessions. Moreover, the fact that the underlying mechanisms of CBT remain poorly understood continues to hinder the use of CBT to treat visceral pain. To unravel the underlying mechanisms of CBT, we and others have employed a rodent model of environmental enrichment (EE), the animal analog of CBT (20). In this review, we discuss the clinical presentation of chronic visceral pain, focusing on IBS. We will also explore the use of both pharmacological and non-pharmacological therapies in treating abdominal pain in IBS patients. We will summarize the most recent research findings on the neuronal and molecular changes that contribute to chronic visceral pain, and discuss the mechanisms underlying the efficacy of behavioral therapies including CBT in ameliorating chronic pain symptoms based on the latest data from experimental models.

## Visceral Pain in IBS

Chronic visceral pain is defined as long-lasting, poorly localized pain emanating from the abdominal region (7, 12) and is a hallmark feature of IBS. Changes in the communication between the central nervous system and the enteric nervous system lead to visceral hypersensitivity and abdominal pain (12). Recent studies suggest that induction and maintenance of visceral hypersensitivity is a multifactorial process that may occur in both the peripheral and central nervous system. Peripherally, infectious agents or altered microbiota content can disrupt the normal functioning of the GI barrier and cause sensitization of nociceptive signals from the enteric nervous system to the brain (13, 14). Growing evidence suggests that psychological and psychosocial factors such as stress act as a key risk factor that triggers or exaggerates IBS symptoms, indicating a role of central regulation in GI function (21). Furthermore, early life stress, such as abuse, is associated with the development of IBS in adulthood (22, 23). Patients with IBS exhibit higher incidence of anxiety (15–45%) or depression (20–30%) (24). IBS patients also displayed significantly higher activity in brain regions involved in stress processing including the amygdala (25, 26). Pre-clinical studies have shown a critical role for corticotropin-releasing hormone (CRH) signaling in the induction of chronic visceral pain. CRH is a potent activator of the hypothalamic-pituitary-adrenal (HPA) axis which regulates the body's response to stress. Increased CRH expression activates the HPA axis leading to a release of cortisol. Under normal conditions, the released cortisol activates glucocorticoid receptors (GRs) in the hypothalamus to inhibit CRH production and reduce HPA activity. However, under chronic stress, persistent cortisol exposure causes alterations in GR signaling that inhibit this negative feedback loop leading to chronic activation of the HPA axis (27). In a female-specific rodent model, unpredictable early life stress uncoupled GR's control of CRH leading to increased CRH expression in the amygdala and increased visceral hypersensitivity (28). Adult stress and anxiety models of IBS use physical stimuli [restraint stress (29, 30), cold exposure (31), and forced swim test (32)] and psychological stimuli such as water avoidance stress (WAS) (27, 33) to induce visceral hypersensitivity. Chronic WAS specifically has been shown to decrease GR expression and increase CRH expression in the central nucleus of the amygdala (CeA) which induces visceral hypersensitivity that persists long after the stressor has been removed (20, 27, 34).

Experimental models of post-inflammatory IBS have revealed that previous inflammatory insult in the colon of rodents sensitizes afferent neurons to induce visceral hypersensitivity mediated by increased expression of nociceptive receptors including calcitonin gene-related peptide (CGRP) (35) and transient receptor potential cation channel subfamily V member 1 (TrpV1) (36) in the dorsal root ganglia cells (37, 38) leading to enhanced nociceptive transmission. Taken together, these studies demonstrate that changes in the processing of sensory signals in the central and peripheral nervous systems contribute to the pathophysiology of chronic visceral pain.

### Pharmacological Therapies for IBS

IBS patients with mild symptoms are advised to make changes in their diet, lifestyle, and to take over-the-counter agents like laxatives and fiber supplements. The FDA has approved a number of pharmacological therapies for use in patients with moderate to severe IBS as shown in Table 1, highlighting its multifactorial etiology. Gastroprokinetics like linaclotide and lubiprostone which act by increasing fluid secretion in the small intestine have been approved for use in IBS-C patients. Linaclotide is a selective agonist of guanylate cyclase C (GC-C), which activates GC-C receptors on intestinal epithelial cells to increase secretion to the lumen (66, 67) Linaclotide is also involved in the modulation of afferent gut nerve activity to affect visceral nociception (66, 68, 69). Lubiprostone is a prostaglandin-derived bicyclic fatty acid, which eases constipation by increasing intraluminal chloride ion secretion, causing a passive influx of water and sodium to increase intestinal peristalsis and colonic laxation (40). Alosetron, cilansetron, and eluxadoline have been FDA-approved for use in IBS-D patients. Alosetron and cilansetron are 5-HT_3_ receptor antagonists that can aid in relaxing the colon and slowing lower GI motility while eluxadoline is a μ-opioid receptor agonist, which decreases GI transit by reducing muscle contractions and fluid secretion in the intestine. Both alosetron and eluxadoline have also been shown to relieve abdominal pain in IBS-D patients (45). Neuromodulators have also shown efficacy in controlling IBS symptoms. Low doses of tricyclic antidepressants such as amitriptyline, desipramine, and nortriptyline can be used to relieve abdominal pain in IBS patients (48–50, 52). Selective serotonin reuptake inhibitors like fluoxetine (52) and serotonin-noradrenergic reuptake inhibitors including duloxetine (53) and venlafaxine (54) are prescribed to IBS patients to attenuate abdominal pain. Delta ligand agents (selective voltage-gated calcium channel agonists) like gabapentin and pregabalin have also been used to treat visceral hypersensitivity and abdominal pain in IBS patients (51). Clinical trials also revealed that rifaximin (an antibiotic) can reduce abdominal pain sensation in adult IBS patients (55). Unfortunately, most of the therapies prescribed for IBS are intended primarily to control symptoms rather than treat the underlying causes. Pharmacological therapies for IBS also present with detrimental side effects. For example, linaclotide and lubiprostone can cause diarrhea in some patients (39, 67, 70), whereas eluxadoline has been reported to cause constipation, nausea, and abdominal pain, and has been associated with pancreatitis (45). The neuromodulators have neurological and mood-altering side effects which adversely affect the patients' quality of life (47, 50). These issues leave many patients with IBS unsatisfied with their current care.

**Table 1:** A summary of the most common classes of pharmacological and non-pharmacological interventions used for controlling irritable bowel syndrome (IBS) symptoms, as well as their therapeutic effects.

	**Classification**	**Intervention**	**Symptom management**
Pharmacological interventions	Gastroprokinetics	Linaclotide (39) Lubiprostone (40)	Increases intestinal fluid secretion and transit in IBS-C (39, 41) Attenuates abdominal pain (40, 42)
	5-HT receptor antagonists	Alosetron (43) Cilansetron (44)	Reduces intestinal fluid secretion and transit in IBS-D (43–46) Attenuates abdominal pain (44–46)
	Opioid receptor agonists	Eluxadoline (45)	
	Tricyclic antidepressants (TCAs)	Amitriptyline (47)	Reduces diarrhea (47), Attenuates abdominal pain (47, 48)
		Desipramine (48, 49)	Reduces abdominal pain (48, 49)
		Nortriptyline (49, 50)	Reduces abdominal pain (49, 50)
	Delta ligand agents	GabapentinPregabalin (51)	Attenuates visceral hypersensitivity (50, 51)
	Selective serotonin reuptake inhibitors (SSRIs)	Fluoxetine	Attenuates abdominal pain Treats mood disorders (52)
	Serotonin–noradrenaline reuptake inhibitor (SNRIs)	Duloxetine (53) Venlafaxine (54)	Decreases abdominal pain (53, 54)
	Antibiotics	Rifaximin	Attenuates pain in IBS patients (55)
Non-pharmacological interventions	Behavioral interventions	Cognitive behavioral therapy	Attenuates abdominal & Somatic pain Improved comorbid mood disorders (including anxiety, depression) (19, 56, 57)
		Gut-directed hypnotherapy Meditation Mindfulness	Attenuates abdominal pain Reduces colonic motility (58–60)
	Alternative therapies	Acupuncture	Attenuates abdominal pain (61, 62), Improves GI motility (62–64)
		Neurostimulation	Decreases abdominal pain in children with IBS (65)

## Emerging Non-Pharmacological Therapies for IBS

Behavioral therapies have historically been used to effectively control mood and anxiety disorders by countering the effects of stress. Because stress reactivity plays an important role in the pathophysiology of chronic visceral pain (7, 71), non-pharmacological interventions have been employed to control IBS symptoms including regular stress management, relaxation and mediation, mindfulness, gut-directed hypnotherapy, and CBT (72). Alternative therapies like acupuncture are also employed to reduce abdominal pain. Auricular neurostimulation therapy is a novel alternative therapy that has been shown to decrease abdominal pain in adolescents (65). CBT, gut-directed hypnotherapy, and acupuncture are the most researched techniques and the most well-known non-pharmacological techniques that have been employed for IBS.

### Gut-Directed Hypnotherapy

Gut-directed hypnotherapy combines body relaxation and mental exercises to influence pain sensation in IBS patients. This requires the patient to be placed in a trance-like state of relaxation. In this state, the patient is given suggestions for how best to improve their IBS symptoms including relaxation and emotional control (73). Gut-directed hypnotherapy typically requires 12 daily sessions to achieve the maximum benefits from the treatment (58) including the ability to distinguish between abdominal sensations and thoughts of abdominal sensations (73). The original study conducted on 30 patients with severe IBS symptoms showed that gut-directed hypnotherapy over a 3-month period significantly improved IBS symptoms, with most patients reporting mild to no symptoms which were sustained long after completion of the treatment (74, 75). Subsequent studies have confirmed these findings (59, 76) and others have also showed that gut-directed hypnotherapy is capable of reversing extra-colonic symptoms of IBS including mood, work attitude, and psychic and physical well-being (77). A long-term study following 200 IBS patients who had been enrolled in a gut-directed hypnotherapy session revealed that most of these patients maintained the benefits of this therapy up to 5 years after their last session (78, 79). Pain relief is not only due to relaxation as studies have shown that specific hypnotic suggestions are usually required for pain relief (78) although that may not always be the case (80). Gut-directed hypnotherapy has been shown to alter emotions, cause reduced colonic motility (81), and normalize pain sensory thresholds in patients who were previously either hypersensitive or hyposensitive (60). Unfortunately, due to the inherent difficulties in creating an animal model for hypnotherapy, the molecular mechanisms underlying gut-directed hypnotherapy have yet to be delineated.

### Acupuncture

Acupuncture involves the insertion of thin needles through skin at acupoints which communicate with specific visceral organs and have been shown to stimulate nerves, muscles, and connective tissue. Clinical trials on the efficacy of acupuncture in treating IBS symptoms have yielded mixed results (61, 63, 82, 83). Multiple clinical and preclinical trials suggest that acupuncture improves the quality of life and reduces pain perception in IBS patients (61, 84). However, other studies suggest that the benefits of acupuncture are due to a placebo response (63, 82). In animal studies, neurogenic inflammatory spots were found to correspond with acupoints in humans. The number and size of these spots were associated with the severity of visceral pain and evidence suggests that stimulating these neurogenic spots is sufficient to attenuate visceral hypersensitivity in rats (85).

### Cognitive Behavioral Therapy

CBT has been shown to be an effective treatment option for IBS and is currently considered the gold standard for psychotherapy (86) with multiple studies showing CBT is capable of relieving IBS symptoms (87–89). CBT is a form of psychosocial intervention that uses behavioral and cognitive psychology to modify behavior, and alter dysfunctional thinking patterns and anxiety states to improve mental health (90). CBT was first pioneered by Beck et al. (91) and is based on the idea that maladaptive cognitive processes contribute to the potentiation of emotional and behavioral problems, and that changing these maladaptive thoughts and behaviors could lead to symptom relief. Different CBT protocols are mild to moderately effective in managing substance abuse such as cannabis and cocaine dependence (92). CBT-based coping skills were helpful in controlling nicotine addiction (93). CBT is also beneficial in controlling persistent psychotic symptoms of schizophrenia such as hallucinations and delusions (94), and there is evidence that CBT in addition to pharmacological approaches may be effective in patients with acute psychosis (95). In patients with depressive disorders, CBT is effective in reducing depressive and anxiety symptoms (96, 97). CBT in conjunction with medication was also more effective in treating chronic depression than CBT or medication alone (56). CBT is also used to treat various anxiety disorders and multiple studies have shown a medium to large effect size of CBT reducing symptoms of social anxiety (98, 99), panic (100), and post-traumatic stress disorders (101, 102). Meta-analysis of behavioral studies for generalized stress in patients found that CBT was one of the most effective therapeutic options in reducing stress (103, 104). CBT has been shown to decrease chronic pain and increase quality of life for patients suffering from fibromyalgia and musculoskeletal pain (57, 105, 106). CBT has also emerged as a prime candidate to attenuate IBS symptoms (89, 107).

Most forms of CBT treatment for IBS typically include a combination of education about the disorder, relaxation, and cognitive restructuring techniques used to address negative thinking patterns, symptom-related anxiety and hypervigilance, and stress reduction techniques based on the premise that if stress causes symptoms, then stress reduction may provide symptom relief (91). More recent iterations of CBT include practicing coping skills, problem-solving skills, de- catastrophizing skills, and exposure therapy (18).

Protocols involving exposure to an enriched environment (EE) have been developed as the rodent analog of CBT and are used to investigate the molecular effects of behavioral interventions in rodents. EE involves exposing animals to a novel environment with sufficient physical and social stimuli to promote cognitive and physiological well-being (108). EE typically uses some combination of physical (toys, platforms, and tunnels) and social cues as well as exercise motivators to simulate a stimulating novel environment (109, 110). EE has been studied extensively in the context of learning and memory where EE promotes hippocampal neuroplasticity and increases neuronal spine density to improve learning and memory (108, 111–113). Animal models of mood and depression disorders also suggest exposure to EE improves recovery from and decreases anxiety-like symptoms due to restraint and social defeat stress via hippocampal (114) and amygdalar (115) dependent mechanisms, respectively. Studies on neuropathic pain revealed that exposure to EE can reduce infant nerve injury-induced anxiety and depressive behavior in adolescent rodents (116). EE also reverses neuropathic pain in rodents (117) while either physical or social EE is sufficient to decrease the recovery time after induction of chronic inflammatory pain in rats (118). A recent study on felines showed that exposure to EE can decrease symptoms of idiopathic cystitis (119). Taken together, these studies demonstrated that EE was capable of reducing stress, anxiety, and visceral pain sensation highlighting a role of behavioral interventions as therapeutic candidates to attenuate stress-induced visceral pain. Our laboratory recently reported that exposing rats to EE 7 days before and 7 days during a WAS protocol persistently inhibited stress-induced visceral and somatic hypersensitivity long after cessation of EE (20). EE prevented the stress-induced decrease in GR expression in the CeA and inhibited CRH-induced potentiation of the stress response to block the development of visceral hypersensitivity (20).

## Discussion

Clinical and preclinical studies clearly show that multiple factors are involved in the pathophysiology of visceral pain which might explain why finding the ideal therapy has been elusive. Current pharmacological treatments available for IBS focus on symptom relief rather than reversing the underlying pathology. Stress, anxiety, and inflammation cause changes in the expression of genes (GR, CRH, and TRPV1) in brain centers critical for stress reactivity and sensory neurotransmission (amygdala, dorsal horn). These alterations ultimately potentiate stress reactivity and sensitize nociceptive afferent fibers leading to visceral hypersensitivity in IBS. Behavioral therapies including hypnotherapy and CBT have already been used extensively as effective behavioral therapies for cognitive, mood, and neuropathic pain disorders and now clinical and pre-clinical trials are showing that behavioral therapies can also ameliorate chronic visceral pain (Table 1). Unfortunately, the lack of trained CBT specialists (120), difficulty of scheduling and maintaining weekly visits to the hospitals for patients (121), and a general misunderstanding of how behavioral therapies work have all contributed to the low adoption rate of CBT for IBS symptoms (122).

**Figure 1:**
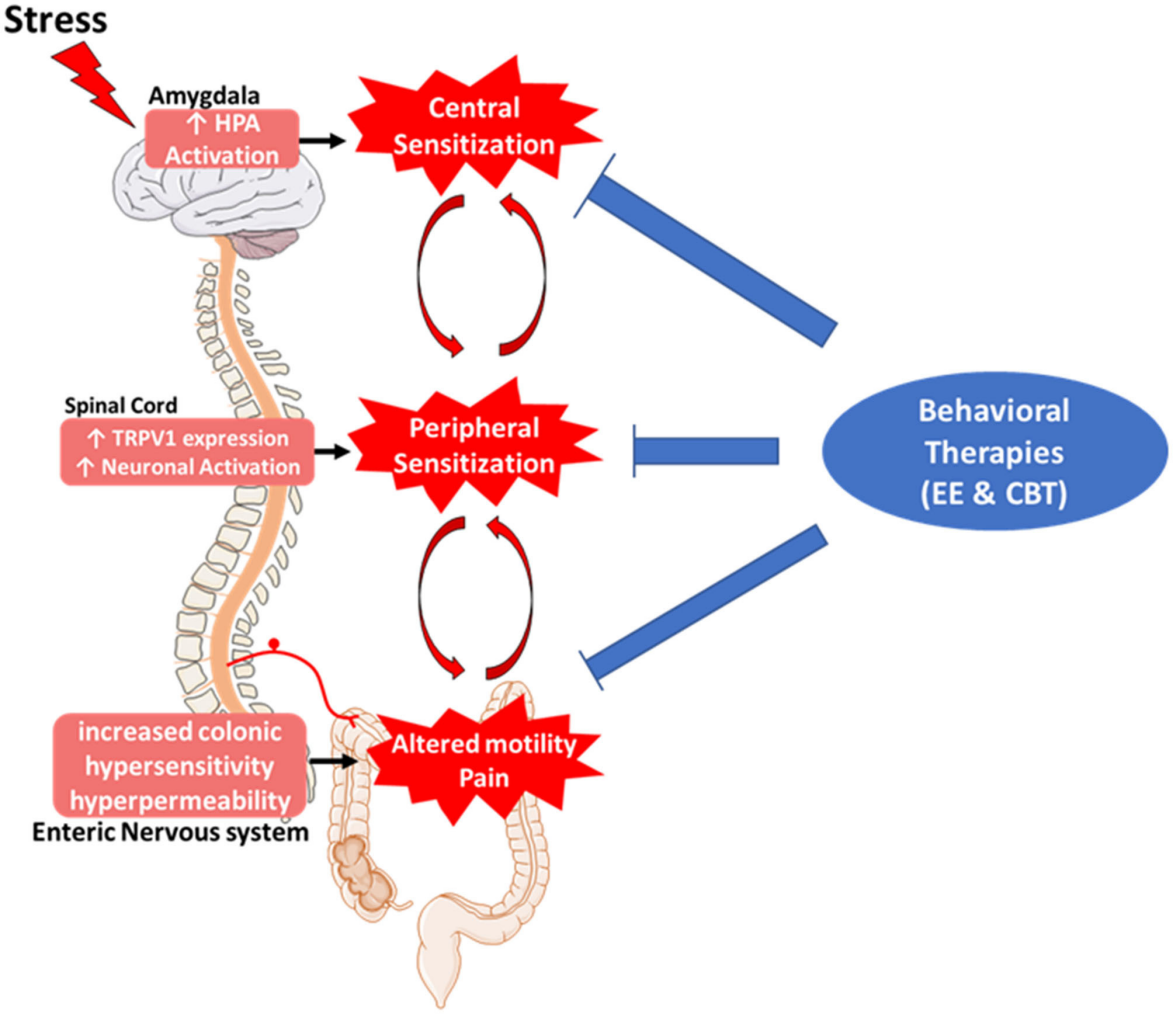
Role of behavioral therapies in the pathophysiology of irritable bowel syndrome (IBS). IBS symptoms can be caused by bidirectional disruptions in the brain–gut axis. Chronic stress-induced hyperactivation of the hypothalamic-pituitary-adrenal axis in the brain leads to increased expression of pro-nociceptive genes and causes sensitization of nociceptive afferents in the spinal cord and enteric nervous systems leading to chronic visceral pain and altered motility. Pharmacological treatments are geared toward ameliorating visceral symptoms without treating the underlying neurological causes. Behavioral therapies show promise in inhibiting visceral pain as well as reversing enhanced stress reactivity and afferent sensitization in the central and peripheral nervous systems, respectively, leading to a more comprehensive and long-lasting relief of IBS symptoms.

In recent years, interest in CBT-based treatment has increased leading to more clinical research and understanding of CBT techniques. Other variations of CBT have been developed to ease the burden of the patients having to constantly visit the clinic for CBT sessions. Studies have shown that patients can be trained to effectively practice CBT techniques remotely. Patients who attended an initial CBT session with a physician and then had subsequent follow-ups via the telephone reported improvements in their IBS symptoms similar to patients who visited a clinician for every session (123). CBT sessions delivered online were also capable of ameliorating IBS symptoms, although the treatment effects were less than with patients who at least one in-person session (124). Preclinical studies using EE have also revealed the molecular mechanisms underlying the effects of behavioral therapies, specifically how EE can reverse stress-induced alterations in the central and peripheral nervous systems to inhibit the development of chronic pain (Figure 1). Gut-directed hypnotherapy and CBT are behavioral therapies for IBS that show efficacy in reversing the underlying causes of the disorder including chronic stress reactivity and enhanced nociception. Other non-pharmacological techniques such as acupuncture have also shown some efficacy in controlling IBS symptoms although with mixed results.

One caveat when interpreting clinical data from behavioral studies is the risk of over-interpretation of placebo effects. Multiple studies have shown significant effects of psychological placebos in behavioral interventions for anxiety and depression (125, 126). It should be noted that the beneficial effects of the placebos were influenced mostly by patient expectations and desires and not the specific placebo (127). Research has also shown that appropriate “placebos” such as in-person meetings and encouragement can be used to improve the effectiveness of non-pharmacological therapies in treating IBS symptoms (126, 128).

In conclusion, non-pharmacological therapies have been shown to induce molecular and psychological changes that quantifiably reverse the pathophysiology of visceral pain in clinical and preclinical studies. Although more research is needed to unravel the mechanisms underlying CBT, experimental models employing EE are being used to determine the role of EE to influence gene expression (20). This review suggests that behavioral interventions could be a very effective therapy either on their own or in combination with other pharmaceutical options in treating IBS and should be offered more widely in IBS treatment programs.

## Disclosure

Research in BG-V laboratory has grant funding from Ironwood Pharmaceuticals, Blue Therapeutics, and TEVA Pharmaceuticals.

## Author Contributions

AO was the lead author in writing the review. TY used her expertise to write certain sections of the main text. BG-V mentored and supervised the team, providing critical input on the scope and topics included in the review. All authors contributed to the article and approved the submitted version.

## Conflict of Interest

The authors declare that the research was conducted in the absence of any commercial or financial relationships that could be construed as a potential conflict of interest.
